# Salinity Stress Acclimation Strategies in *Chlamydomonas* sp. Revealed by Physiological, Morphological and Transcriptomic Approaches

**DOI:** 10.3390/md22080351

**Published:** 2024-07-29

**Authors:** Chiara Lauritano, Emma Bazzani, Eleonora Montuori, Francesco Bolinesi, Olga Mangoni, Gennaro Riccio, Angela Buondonno, Maria Saggiomo

**Affiliations:** 1 Ecosustainable Marine Biotechnology Department, Stazione Zoologica Anton Dohrn, Via Acton, 80133 Naples, Italy; eleonora.montuori@szn.it; 2 Research Infrastructure for Marine Biological Resources Department, Stazione Zoologica Anton Dohrn, Villa Comunale, 80121 Naples, Italy; bazzanie@tcd.ie (E.B.); angela.buondonno@szn.it (A.B.); maria.saggiomo@szn.it (M.S.); 3 Smurfit Institute of Genetics, School of Genetics and Microbiology, Trinity College Dublin, College Green, Dublin 2, D02 VF25 Dublin, Ireland; 4 Department of Chemical, Biological, Pharmaceutical and Environmental Sciences, University of Messina, Viale F. Stagno d’Alcontres 31, 98166 Messina, Italy; 5 Department of Biology, University of Naples Federico II, Via Cinthia 21, 80126 Naples, Italy; francesco.bolinesi@unina.it (F.B.); olga.mangoni@unina.it (O.M.); 6 CoNISMa, Piazzale Flaminio, 9, 00196 Roma, Italy; 7 Department of Biology and Evolution of Marine Organisms, Stazione Zoologica Anton Dohrn, Villa Comunale, 80121 Naples, Italy; gennaro.riccio@szn.it

**Keywords:** *Chlamydomonas*, salinity stress, transcriptome analysis, photosynthetic efficiency, growth rate

## Abstract

Climate changes may include variations in salinity concentrations at sea by changing ocean dynamics. These variations may be especially challenging for marine photosynthetic organisms, affecting their growth and distribution. *Chlamydomonas* spp. are ubiquitous and are often found in extreme salinity conditions. For this reason, they are considered good model species to study salinity adaptation strategies. In the current study, we used an integrated approach to study the *Chlamydomonas* sp. CCMP225 response to salinities of 20‰ and 70‰, by combining physiological, morphological, and transcriptomic analyses, and comparing differentially expressed genes in the exponential and stationary growth phases under the two salinity conditions. The results showed that the strain is able to grow under all tested salinity conditions and maintains a surprisingly high photosynthetic efficiency even under high salinities. However, at the highest salinity condition, the cells lose their flagella. The transcriptomic analysis highlighted the up- or down-regulation of specific gene categories, helping to identify key genes responding to salinity stress. Overall, the findings may be of interest to the marine biology, ecology, and biotechnology communities, to better understand species adaptation mechanisms under possible global change scenarios and the potential activation of enzymes involved in the synthesis of bioactive molecules.

## 1. Introduction

Changes in environmental salinity levels present a dual challenge for photosynthetic organisms by causing both ionic and osmotic stress, due to either high or low salinities. Additionally, salt stress can cause the production of reactive oxygen species (ROS), threatening not only the growth but also the survival of the organism. This makes salinity one of the most important abiotic factors affecting the growth and distribution of photosynthetic organisms worldwide. Marine ecosystems are particularly vulnerable to salinity changes, and understanding how organisms adapt to these changes is crucial for predicting the potential impacts of climate change on marine communities. Moreover, the anticipated alterations in global salinity patterns due to anthropogenic climate change [1] are expected to have strong consequences on the distribution and composition of microalgal communities. This study is pivotal in advancing our understanding of the adaptive mechanisms of green microalgae and in developing strategies for mitigating the effects of salinity stress on marine and agricultural systems [2]. To withstand environmental fluctuations and extreme conditions, marine organisms have evolved many mechanisms to maintain cellular homeostasis [3,4,5,6]. Green microalgae, which are important primary producers in many different environments, including salt lakes, estuaries, and brine channels, often face high and fluctuating salt concentrations. To cope with these extreme conditions, they exhibit simultaneous morphological, physiological, and molecular adjustments that enhance cell survival in the presence of salt stress [7].

Among microalgae, the genus *Chlamydomonas* has emerged as a valuable model for studying the physiological and molecular responses to salinity stress, together with other known salt-tolerant species like *Dunaliella salina* [7,8]. Unicellular green algae from the genus *Chlamydomonas* possess great plasticity and adaptability to most abiotic stresses [9], they are capable of growing in both fresh and saline water environments, facing extreme salinity variations [10]. It has been shown that phenotypic plasticity can arise from multiple underlying molecular mechanisms, but many of them rely on gene expression changes [11,12]. The plastic regulation of genes involved in glycerol synthesis is known to be key in *Dunaliella salina* response salinity changes, together with changes in cell volume, and intracellular ions concentration [13]. Moreover, a high physiological plasticity is often manifested in the functioning of the photosynthetic apparatus and in the pigment composition [14,15]. 

*Chlamydomonas* spp. have been widely used as model organisms in both basic and applied research, with *C. reinhardtii* being the most studied species of the genus [7], with sequenced nuclear, mitochondrial, and chloroplast genomes [16]. Additionally, for various *Chlamydomonas* species, both the genome and transcriptome sequences have been published, making genetic modifications possible via genome-editing methods [17,18,19,20,21]. The straightforward genetic manipulation and easy cultivation make this species particularly amenable to classic genetic analyses, and to study algal responses to stressful environmental conditions [9], offering a convenient and well-established model for investigating potential solutions to salinity-related issues. Carrasco Flores et al. [22] highlighted in their work how the marine specie *Chlamydomonas* sp. SAG25.89 may be a possible model organism for marine microalgae, being closely related to the freshwater *C. reinhardtii*. *Chlamydomonas* sp. SAG25.89 can tolerate a wide range of salinities, making it an excellent model for understanding osmoregulation mechanisms. Furthermore, Carrasco Flores et al. [22] also sequenced the nuclear genome of *Chlamydomonas* sp. SAG25.89. The GC content in the genome makes reporter genes and selection markers usable in both organisms, both the freshwater *C. reinhardtii* and marine *Chlamydomonas* sp. [22]. 

In this study, we aim to explore the effects of salinity stress on an estuarine *Chlamydomonas* species (*Chlamydomonas* sp. CCMP225, https://ncma.bigelow.org/CCMP225 accessed on 17 July 2024), investigating the alterations in growth patterns, physiological parameters, and molecular responses. By employing a multidisciplinary approach integrating physiological, microscopy, and molecular techniques, we seek to unravel the intricate mechanisms employed by *Chlamydomonas* to adapt and survive under salinity stress. This research could help elucidate the potential implications of salinity stress on the overall ecology and productivity of marine environments.

## 2. Results

### 2.1. Growth Curves and Morphological Observations

**Figure 1:**
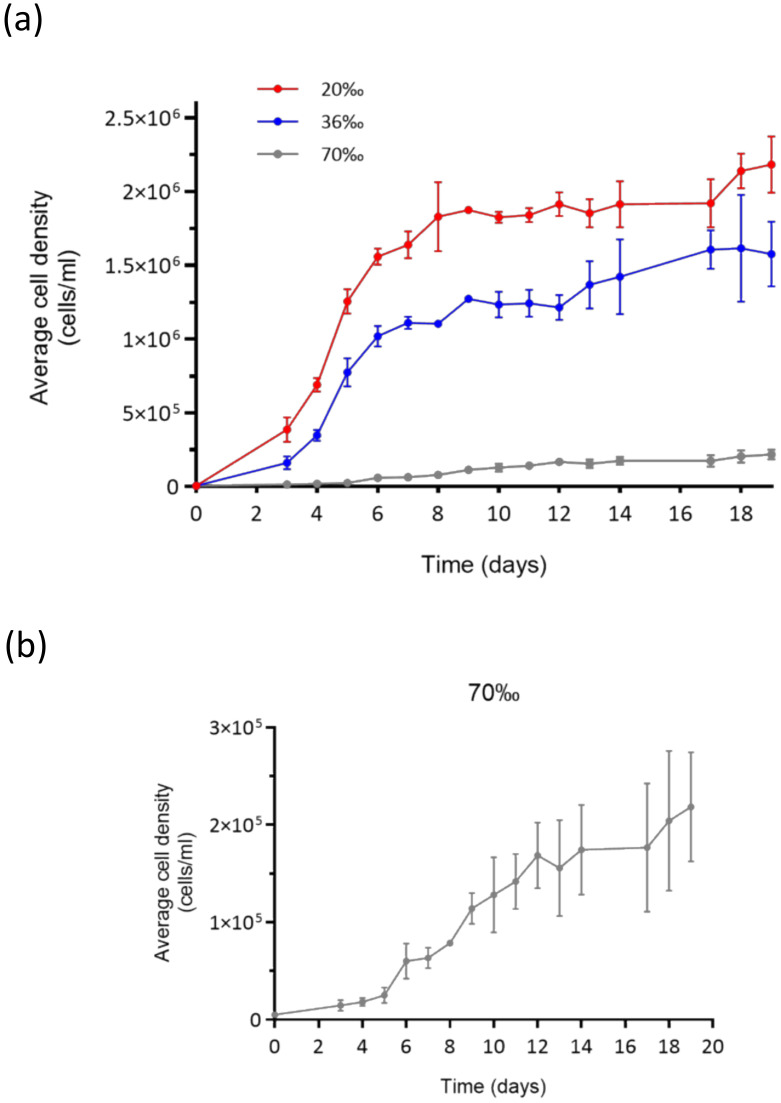
Growth curves of *Chlamydomonas* sp. under the three salinity conditions: 20‰ (red line), 36‰ (blue line), and 70‰ (grey line). The curves represent mean values of three replicates with standard deviation bars: (**a**) the three conditions together, and (**b**) a close-up on the growth curve at salinity 70‰.

**Figure 2:**
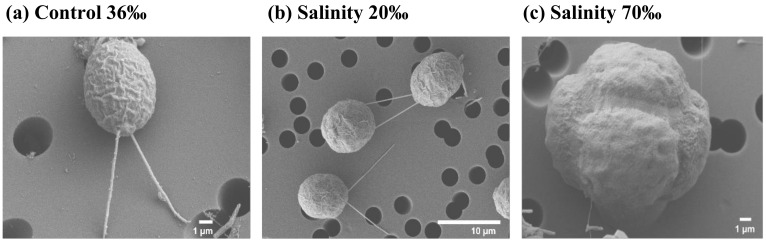
Morphological analysis at the scanning electron microscope (SEM) of *Chlamydomonas* sp. cultured in: (**a**) control condition, salinity 36‰; (**b**) salinity 20‰; and (**c**) salinity 70‰.

Cell counts showed that the cells grew under all experimental conditions (Figure 1a). In particular, the growth curves at 36‰ and 20‰ presented a very similar trend, with a growth rate of 1.3 division/day (div/day), and 1.4 div/day, respectively. From Day 3, we observed an immediate increase in cell density, up to approximately 19 × 10^4^ at 36‰, and 39 × 10^4^ at 20‰, to reach a maximum of 1.3 × 10^6^ and 1.9 × 10^6^ at Day 9. From Day 10 to Day 20, in both conditions, a stationary phase persisted. Surprisingly, the microalgae grown at 70‰ salinity were not only surviving, but also growing, with a growth rate, approaching 0.5 div/day (Figure 1b). In fact, from Day 3, we can observe a slow increase of cell density, with 18 × 10^3^ cells/mL. The cell density slowly increased until Day 20, when the experiment was concluded. Morphological analyses at the scanning electron microscope (SEM; Figure 2) showed no evident differences between the microalgae grown at 20‰ and 36‰. On the other hand, most of the cells grown at 70‰ appear to have lost their flagella, and many of them were resembling a palmelloid state. 

### 2.2. Photosynthetic Efficiency

**Figure 3:**
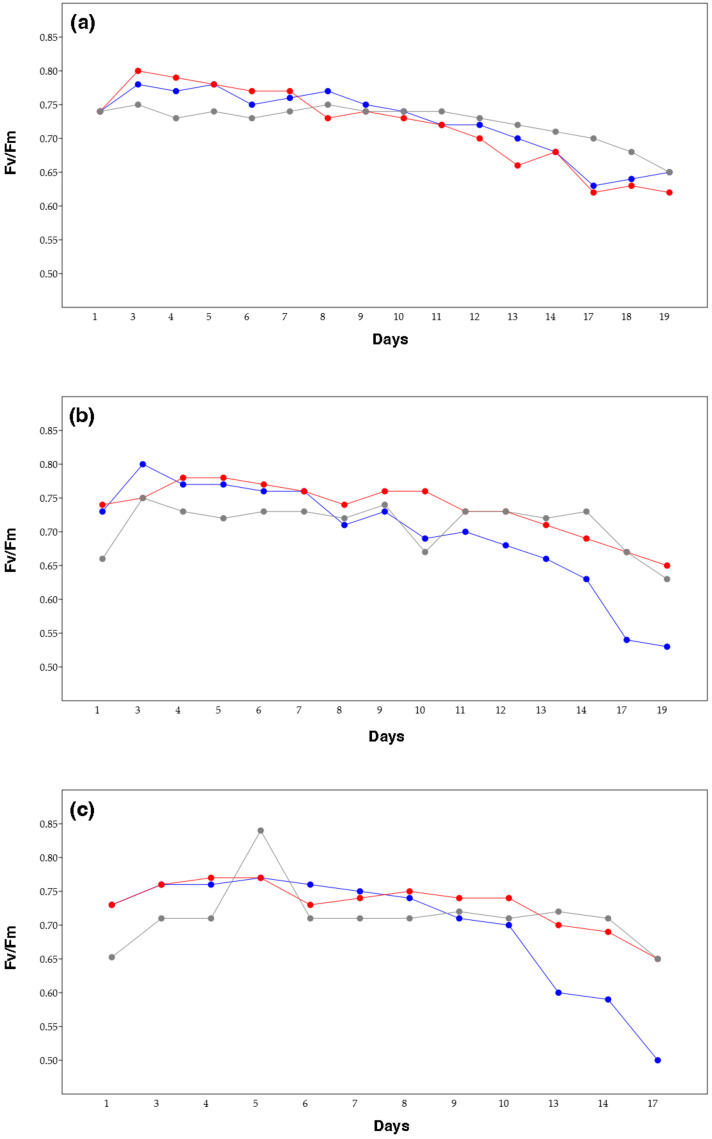
Fv/Fm values of *Chlamydomonas* sp. in the studied salinity conditions: 20‰ (red line), 36‰ (blue line), and 70‰ (grey line), in three different biological replicates: (**a**) experiment I; (**b**) experiment II; and (**c**) experiment III.

The maximum photochemical efficiency of photosystem II (Fv/Fm) has been widely used to assess the impact of environmental stress on phytoplankton. The Fv/Fm values were measured daily, during the entire duration of the experiment. These values showed slightly different trends, depending on the salinity, and on the replicate, but no strong variations were present (Figure 3). In general, the results indicated a high maximum quantum efficiency (~0.7), with the samples grown at 36‰ being characterized by a decreasing trend towards the end in all the three replicates, while the samples grown at 20‰ and 70‰ showed lower fluctuations. Specifically, in the first biological replicate, experiment I (Figure 3a), samples grown at 20‰ and 70‰ showed a similar trend, with values decreasing from day 3 (0.80 and 0.75, respectively) to day 17 (0.63 and 0.70, respectively). The samples grown at 36‰ showed a value of 0.75 ± 3 until day 12, after which the photosynthetic efficiency slowly decreased to 0.65 at day 19. In the second biological replicate, experiment II (Figure 3b), Fv/Fm ranged between 0.78 and 0.65 in the samples grown at 20‰, displaying a decreasing trend after day 11. The samples grown at 70‰ showed rather similar values, 0.73 ± 2 (except a spike at day 10) until day 14, and, after that, decreasing to 0.63 at day 19. Samples grown at 36‰ ranged between 0.80 (day 3) and 0.53 (day 19). In the third biological replicate, experiment III (Figure 3c), the lower salinity samples (20‰ and 36‰) showed similar values (0.75) until day 8, after which the values of the cells grown at 36‰ decreased to 0.5 (day 17), while 20‰ reached 0.65 (day 17). The 70‰ sample was characterized by a low variation of values, ranging between 0.71 and 0.65, except for a spike of 0.81 (day 5).

### 2.3. Sequencing

To sequence and identify expressed genes in the *Chlamydomonas* sp. (CCMP225) samples derived from different salinity conditions (six samples at salinity 20‰, of which there was a triplicate in the exponential phase and a triplicate in the stationary phase, and six samples at salinity 70‰, of which there was a triplicate in the exponential phase and a triplicate in the stationary phase), we used RNA-sequencing by Illumina NovaSeq6000. Prior to further analysis, a quality check was performed on the sequencing data. The Bioinformatic analysis involved several steps: the quality check of the reads, removal of the adapters and very short reads, reads alignment, and transcriptome expression quantification analysis. The quality control of the reads, used to check the quality of the raw data sequencing based on statistics, was performed by the FastQC tool available on [18]. The probability of identifying a base incorrectly equal to 0.1 (10%), 0.01 (1%), and 0.001 (0.1%) produce, respectively, a value of phred score (q or Q) of 10, 20, and 30.

The adapter is ligated to the end of each molecule during library preparation. Consequently, the output reads contained the sequence of the molecule of interest and the adapter sequence. Thus, the resulting reads may contain a significant part of the adapter. Essential first tasks during the analysis of such data were, therefore, to find the reads-containing adapters and to remove the adapters when they occurred. For this purpose, the bioinformatic tool cutadapt (version 2.5) [23] was used to remove the adapter sequence (if present) and the very short reads (reads length < 25). 

High-quality reads from the samples were used as input to perform transcriptome assembly, with Trinity [23]. Then, the estimation of the transcript abundance was performed, considering each sequenced sample via the RSEM methods [24]. The raw assembled transcriptome included 164,273 transcripts grouped in 104,775 genes (Table 1). The mean GC content was 52.24%. For the transcripts, the average and median contig length were 996.53 bp and 488 bp, respectively. The N50 was 1945 bp.

**Figure 4:**
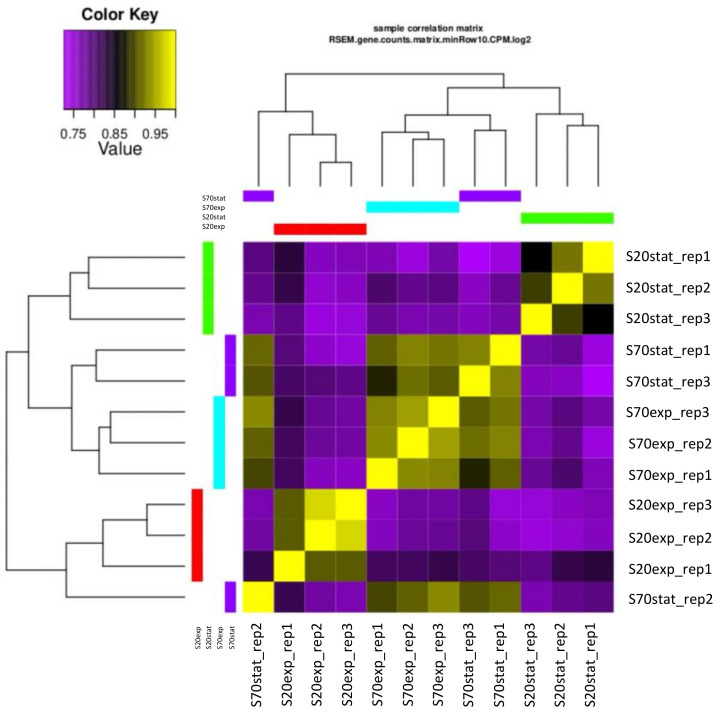
Sample correlation matrix of all genes in the samples. The abbreviation exp stands for exponential and stat for stationary.

To ensure that the biological replicates are well-correlated, and to investigate the relationships among the samples, we performed the principal component analysis (PCA; Supplementary Figure S1) and the correlation analysis of samples (Figure 4).

### 2.4. Differential Gene Expression Analysis

**Figure 5:**
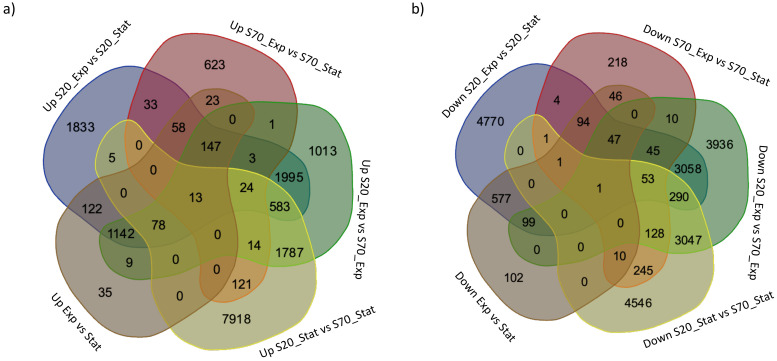
Venn diagrams showing numbers of up- (**a**) and down-regulated (**b**) differentially expressed genes (DEGs) different and shared among the experimental setups: exponential (exp) and stationary (stat) growth phases of microalgae cultivated at 20‰ and 70‰ salinity (S).

To identify differentially expressed genes in the different culturing conditions, we used the Trinity DeSeq2 package [25], between: 1) salinity 20‰ (S20)_Exp vs. S20_Stat, 2) salinity 70‰ (S70)_Exp vs. S70_Stat, 3) S20_Exp vs. S70_Exp, 4) S20_Stat vs. S70_Stat, and 5) Exp vs. Stat. Table 2 displays the number of differentially expressed genes (DEGs) identified with a false discovery rate (FDR) ≤0.05 in total, and the up-regulated (FC ≥ 1.5) or down-regulated (FC ≤ −1.5) genes. Specifically, there were 6036 up-regulated DEGs and 9040 down-regulated DEGs comparing S20_Exp vs. S20_Stat, 1060 up-regulated DEGs and 903 down-regulated DEGs comparing S70_Exp vs. S70_Stat, 6809 up-regulated DEGs and 10704 down-regulated DEGs comparing S20_Exp vs. S70_Exp, 10,543 up-regulated DEGs and 8322 down-regulated DEGs comparing S20_Stat vs. S70_Stat, and 1627 up-regulated DEGs and 977 down-regulated DEGs comparing Exp vs. Stat. These results showed that the numbers of DEGs were higher when comparing the two salinity conditions, rather than the exponential and stationary phases within the same salinity condition. Similarities or differences among the different experimental setups are shown by the Venn diagrams below (Figure 5). In addition, the DEGs for each comparison and their annotation, when available, are reported in Supplementary Tables S1–S5. 

### 2.5. Functional Annotation

**Figure 6:**
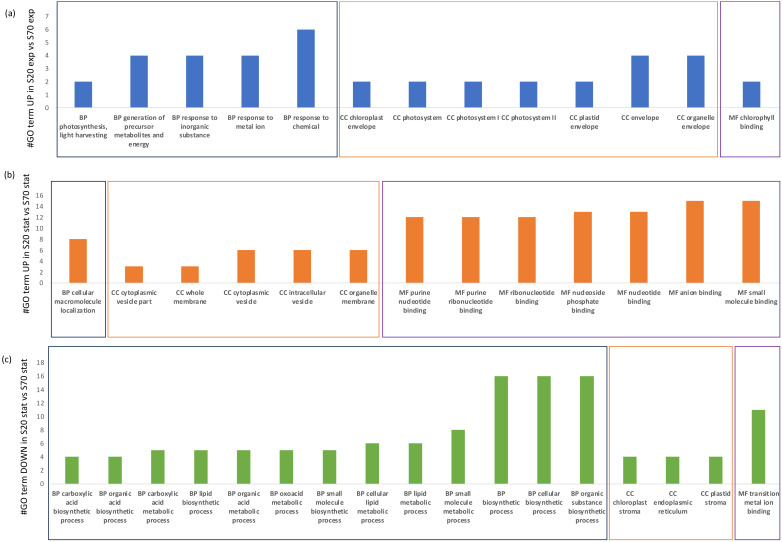
GO terms enriched: (**a**) UP in S20_Exp vs. S70_Exp, (**b**) UP in S20_Stat vs. S70_Stat, and (**c**) DOWN in S20_Stat vs. S70_Stat. The abbreviation GO stands for gene ontology, BP for biological processes (grouped in each panel by a black rectangle), CC for cellular components (grouped in each panel by an orange rectangle), and MF for molecular functions (grouped in each panel by a purple rectangle). Exp stands for exponential and Stat for stationary.

Gene ontology (GO) enrichment (Table 3 and Figure 6) was performed by using Trinotate and GOseq. The analysis was performed on genes differentially expressed that exceed the FDR and fold-change thresholds (FDR *≤* 0.05, |FC| *≥* 1.5). We used Trinotate to generate an annotation report, which includes top-matching blast matches from SwissProt and any corresponding gene ontology (GO) assignments from the TrEMBL/SwissProt databases. Then, the Bioconductor package GOseq was used to perform functional enrichment tests. The GOseq analysis was based on all differentially expressed DEGs and up/down-regulated genes separately.

Within the enriched GO terms for the comparison S20_Exp vs. S20_Stat, there were 33 GO terms considering the up-regulated DEGs and 1 considering the down-regulated DEGs. There were no enriched GO terms in the S70_Exp vs. S70_Stat comparison, while, for S20_Exp vs. S70_Exp, there were 13 enriched GO terms for the up-regulated DEGs. Finally, for the S20_Stat vs. S70_Stat comparison, there were 13 GO terms for the up-regulated DEGs and 17 for the down-regulated DEGs.

In order to better understand the differences induced by salinity stress, we have summarized in Figure 6 the GO categories, biological processes (BP), cellular components (CC), and molecular functions (MF), for the comparisons S20_Exp vs. S70_Exp and S20_Stat vs. S70_Stat. In particular, for the up-regulated DEGs in S20_Exp vs. S70_Exp, the highly represented GO terms were a response to the chemical as BP, organelle envelope and envelope for CC, and chlorophyll binding as MF. For the up-regulated DEGs in S20_Stat vs. S70_Stat, there were cellular macromolecule localization as an enriched BP GO term, an organelle membrane and intracellular vesicle for CC, and small molecule binding and anion binding as MF. Finally, for the down-regulated DEGs in S20_Stat vs. S70_Stat, there were an organic substance biosynthetic process, cellular biosynthetic process, and biosynthetic process as BP, plastid stroma, endoplasmic reticulum, and chloroplast stroma as CC, and transition metal ion binding as MF.

### 2.6. Genes Most Up- or Down-Regulated in S20_Exp vs. S20_Stat

From the differential gene expression analysis between *Chlamydomonas* sp. cells grown at 20‰ salinity, collected in the exponential phase vs. stationary phase, the five most up-regulated DEGs were: the transcripts 3-phosphoshikimine 1 carboxyl vinyl transferase, carbonic anhydrase II, UPF0225 protein VV1_2912, pyruvate dehydrogenase E1 component subunit beta-3 transcript, and Chlorophyll a-b binding protein CP29.2 (Table 4). The most up-regulated gene corresponded to the transcript coding for enzyme3-phosphoshikimine 1 carboxyl vinyl transferase. This enzyme is part of the group of alkyl and aryl transferases, a class of enzymes that catalyzes the transfer of alkyl or related groups. The 3-phosphoshikimine 1 carboxyl vinyl transferase is part of the shikimate pathway and catalyzes the transfer of the enolpyruvyl moiety of phosphoenolpyruvate-PEP to the 5hydroxyl of the shikimate-3-phosphate, to produce enolpyruvyl shikimate 3-phosphate and inorganic phosphate (https://pubchem.ncbi.nlm.nih.gov/substance/381121447 accessed on 30 November 2023). The shikimate pathway is a metabolic pathway involved in the biosynthesis of the amino acids tyrosine, threonine, and tryptophan [26]. The pathway comprises seven steps, which involve seven enzymes, including 3-phosphoshikimine 1 carboxyl vinyl transferase, involved in the synthesis of chorismate. Chorismate, also known as chorismic acid, is the precursor of aromatic amino acids and of aromatic secondary metabolites such as alkaloids and vitamins (i.e., vitamine K and vitamine B9). Alkaloids are natural organic compounds with a large range of bioactivities such as anti-diabetes, anti-asthma, anti-malaria, anti-cancer [27,28], antioxidant, and anti-inflammatory properties [29]. There are some examples of alkaloids approved by the Food and Drug Administration (FDA) for the treatment of cancer: Vinca alkaloids (http://www.ncbi.nlm.nih.gov/pmc/articles/PMC3883245/ accessed on 30 November 2023), Taxanes [30,31,32], Camptothecin [33], Homoharringtonine [34,35], Tetrahydroisoquinoline [36], and Purine alkaloids [37]. 

The second most up-regulated transcript in the differential expression gene analysis of S20_Exp vs. S20_Stat was carbonic anhydrase II, a member of a large family of enzymes involved in the interconversion of carbon dioxide and water to bicarbonate and hydrogen ions. This enzyme has a crucial role in photosynthesis [38]. The UPF0225 protein VV1_2912 codifies for proteins belonging to the UPP0225 family (https://www.uniprot.org/uniprotkb/Q8D8Q7/entry accessed on 13 July 2024). The pyruvate dehydrogenase E1 component subunit beta-3 transcript was also up-regulated. This transcript codifies for the first enzyme involved in the pyruvate dehydrogenase (PDH) complex, named pyruvate dehydrogenase, that catalyzes the conversion of pyruvate in acetyl CoA and CO_2_ (AlphaFold Protein Structure Database (ebi.ac.uk)). The PDH complex is a multienzyme complex that contains E1, E2 dihydrolipoamide acetyltransferase (E2), and lipoamide dehydrogenase (E3) [39]. This complex is involved in the control of the entry of carbon into the mitochondrial tricarboxylic acid cycle to enable cellular energy production [39] (https://www.uniprot.org/uniprotkb/Q2QM55/entry accessed on 28 November 2023). Moreover, the transcript Chlorophyll a-b binding protein CP29.2 was up-regulated. It codifies for the Chlorophyll a-b binding protein CP29.2, involved in the light harvesting and photosynthetic reactions in which it mediates the transition from state I to state II. The transition state determines the affinity of the light harvesting complex (LHC) for photosystem I (PSI) and photosystem II (PSII). The LHC is a light receptor that captures and delivers excitation energy to photosystems (https://www.uniprot.org/uniprotkb/Q93WD2/entry accessed on 28 November 2023).

The five most down-regulated genes were: two transcripts coding for adenylate cyclase, peptide-N(4)-(N-acetyl-beta-glucosaminyl) asparagine amidase, ubiquitin carboxyl-terminal hydrolase 24, and serine/threonine-protein phosphatase 6 regulatory ankyrin repeat subunit C. Adenylate cyclase, also known as adenylyl cyclase, is a key component of the cAMP signaling pathway, responsible for the production of the second messenger adenosine 3′,5′-monophosphate (cAMP) [40], and controls the physiology of the cells, tissues, organs, and organisms in health and diseases [40]. Peptide-N(4)-(N-acetyl-beta-glucosaminyl) asparagine amidase is also known as PNGase (peptide:N-glycanase). It is a cytosolic glycosylation enzyme which catalyzes the non-lysosomal hydrolysis of an N(4)-(acetyl-β-d-glucosaminyl) asparagine residue (Asn, N) into a N-acetyl-β-d-glucosaminyl-amine and a peptide containing an aspartate residue (Asp, D) [41,42]. The transcript ubiquitin carboxyl-terminal hydrolase 24 catalyzes for the ubiquitin-specific protease that regulates cell survival (https://www.uniprot.org/uniprotkb/Q9UPU5/entry; accessed on 30 November 2023). In general, the serine/threonine-protein phosphatases are highly conserved enzymes in eukaryotic evolution. They differ in the various types of interaction between their catalytic subunits with regulatory proteins [43]. Protein phosphatase 6 (PP6) holoenzyme is a heterotrimeric complex formed by the catalytic subunit, a SAPS domain-containing subunit (PP6R), and an ankyrin repeat-domain containing regulatory subunit (ARS) (https://www.uniprot.org/uniprotkb/Q8NB46/entry; https://pubchem.ncbi.nlm.nih.gov/protein/Q8NB46 accessed on 14 January 2024).

### 2.7. Genes Most Up- or Down-Regulated in S20_Exp vs. S70_Exp

From the differential expression analysis of the samples collected during the exponential growth phase under the two different salinities, 20‰ and 70‰, there were three chloroplastic transcripts: Chlorophyll a-b binding protein CP29.2, Chlorophyll a-b binding protein type member F3, and 3-phosphoshikimate 1-carboxyvinyltransferase, chloroplastic (Table 5). The chlorophyll a-b binding proteins form a large family of polypeptides that are encoded by nuclear genes and are synthesized on cytoplasmatic ribosomes, imported across the two membranes of the chloroplast envelope and inserted in the thylakoid membranes [44]. The 3-phosphoshikimate 1-carboxyvinyltransferase, carbonic anhydrase 2, and UPF0225 protein VV1_2912 were also up-regulated in the previous comparison (Section 2.6). 

The five most down-regulated transcripts were serine/threonine-protein kinase fray 1, serine/threonine-protein kinase OSR1, isoprenyl transferase, dehydrodolichyl diphosphate synthase 2, and nitric oxide synthase. The transcripts serine/threonine-protein kinase fray 1 (https://www.uniprot.org/uniprotkb/Q54XL6/entry; accessed on 1 February 2024) and serine/threonine-protein kinase OSR1 codify for two serine/threonine-protein kinases. The latter, also called OXSR1, codifies for an oxidative stress responsive kinase, that regulates the downstream kinases in response to the environmental stress (https://www.ncbi.nlm.nih.gov/gene/9943; accessed on 1 February 2024). Isoprenyl transferase is known to catalyze the condensation of isopentenyl disphosphate (IPP) with allylic phyrophosphates, generating different types of terpenoids (https://www.uniprot.org/uniprotkb/Q97SR4/entry; accessed on 1 February 2024). Dehydrodolichyl diphosphate synthase 2 codifies for a protein that is involved in the pathway of protein glycosylation (https://www.uniprot.org/uniprotkb/Q56Y11/entry; accessed on 1 February 2024). Nitric oxide synthase is a protein that, stimulated by calcium/calmodulin, catalyzed the conversion of L-arginine to L-citrulline, producing nitric oxide (NO) (https://www.uniprot.org/uniprotkb/Q27571/entry; accessed on 1 February 2024).

### 2.8. Genes Most Up- or Down-Regulated in S20_Stat vs. S70_Stat Comparison

The first five up-regulated transcripts found with the DEG’s analysis comparing the stationary phases at the two different salinities (20‰ and 70‰) were: probable inactive leucine-rich repeat receptor kinase XIAO, adenylate cyclase, Adenylate cyclase, Cys-loop ligand-gated ion channel, and Intraflagellar transport protein 22 (Table 6).

The first up-regulated gene was the probable inactive leucine-rich repeat receptor kinase XIAO that is probably involved in the regulation of many cell cycle genes. It is also involved in the regulation of the mediated brassinosteroid signaling pathway (https://www.uniprot.org/uniprotkb/G9LZD7/entry; accessed on 29 January 2024). The enzyme adenylate cyclase can activate other proteins in the cells by phosphorylating them [45], and it is known to have a key regulatory role in the cell. This enzyme is strongly interconnected with the receptors of transmembrane G proteins, integrating the positive and negative signals from the extracellular environment and determining the regulation of cAMP levels in the cell [45]. Finally, the transcript intraflagellar transport protein 22 codifies for intraflagellar transport 22 that is involved in the pathway of biogenesis and maintenance of the organelle (https://www.uniprot.org/uniprotkb/Q9H7X7/entry; accessed on 29 January 2024). Intraflagellar transport 22 is a small GTPase-like component of intracellular transport complex B (https://www.uniprot.org/uniprotkb/Q9H7X7/entry; accessed on 29 January 2024). In general, intraflagellar transport plays essential roles in the assembly and function of flagella [46] (https://www.uniprot.org/uniprotkb/Q9H7X7/entry; accessed on 14 June 2024).

The five most down-regulated transcripts were: serine/threonine-protein kinase fray 1, serine/threonine-protein kinase OSR1, isoprenyl transferase, dehydrodolichyl diphosphate synthase 2, and beta-carotene 3-hydroxylase, chloroplastic. The first four down-regulated transcripts were the same for the comparison between S20_Exp vs. S70_Exp. The fifth down-regulated transcript was the beta-carotene3-hydroxilase, chloroplastic which codifies for the beta-carotene hydroxylase protein that is involved in the biosynthesis of xantophylls (https://www.uniprot.org/uniprotkb/O49815/entry; accessed on 1 February 2024).

### 2.9. Genes Most Up-Regulated in Exp vs. Stat Comparison

Considering the comparison Exp vs. stat, which is a comparison between all the transcripts expressed in the exponential phase vs. stationary phase independently from the salinity condition (Table 7), there were not genes significantly down-regulated. Among the up-regulated genes, there were the transcripts carbonic anhydrase 2,3-phosphoshikimate 1-carboxyvinyltransferase, chloroplastic, Chlorophyll a-b binding protein CP29.2, chloroplastic, UPF0225 protein VV1_2912, and Pyruvate dehydrogenase E1 component subunit beta-3, chloroplastic which were also up-regulated in the previous comparisons, namely, S20_Exp vs. S20_Stat and S20_Exp vs. S70_Exp.

### 2.10. Reverse-Transcription–Quantitative PCR (RT-qPCR) Analyses on Selected Genes

We selected five genes, between the most up- and down-regulated DEGs in the various comparisons as genes of interest (GOI) for the transcriptome validation by RT-qPCR. The results supported the RNA-seq DEG analyses. In fact, the five selected transcripts had the same up- or down-regulation patterns as in the DEG’s results. As reported in Supplementary Table S6, AC, down-regulated in the DEG comparison S20_Exp vs. S20_Stat, was also down-regulated in the RT-qPCR analysis of the −2.781 log2 x-fold ratio. Similarly, Chlorophyll a-b binding protein type member F3, chloroplastic (CABF3), probable inactive leucine-rich repeat receptor kinase XIAO (XIAO), adenylate cyclase (AC), and Carbonic Anhydrase 2 (CA), which were up-regulated in various DEG comparisons (see details in Supplementary Table S6), were also up-regulated by the RT-qPCR of +2.005, +22.482, +23.454, and +1.215 log2 x-fold expression ratio, respectively.

## 3. Discussion

**Figure 7:**
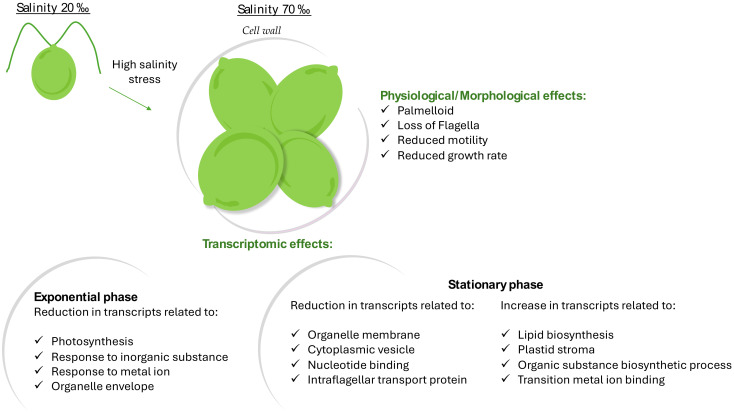
Schematic diagram of main physiological, morphological, and transcriptomic results observed at high salinity.

Considering that salinity is among the major environmental drivers affecting microalgae, and that the global salinity patterns are expected to change due to climate changes, studies on cellular responses to salinity stress are fundamental to predicting how the growth, distribution, and composition of microalgal communities will be affected. As reviewed in a recent paper by our group, numerous studies have focused on understanding the physiological and molecular responses of various species of the *Chlamydomonas* genus to salinity stress [7]. The most common features are a slower growth rate, lowered photosynthetic efficiency, and induced production of mucus at high salinity. In the current cultivation experiment, *Chlamydomonas* sp. CCMP225, an estuarine species, showed a similar trend at 20‰ and 36‰ salinities, with a rapid and immediate growth. On the other hand, at higher salinities (70‰), the cells showed a lower growth rate and a delayed exponential phase, preceded by a longer lag phase. Nonetheless, at 70‰, the cells were not damaged and showed growth even under stressful conditions, maintaining high Fv/Fm levels, but exhibiting a visible reduced motility and flagellar loss. *Chlamydomonas* spp. exposed to certain salt stress agents forms multicellular aggregates known as “palmelloids”, characterized by the loss of flagella [47]. In line with the literature upon stress exposure, we also found a palmelloid state at high salinity, a cluster of 4 to 16 cells surrounded by a cell wall resulting from the division of a single cell (Figure 7) [48,49]. The physiological, morphological, and transcriptomic main results are summarized in Figure 7.

The Fv/Fm data indicated that this species showed a high photosynthetic efficiency under all the different salinity conditions, revealing a high photosynthetic plasticity under high salinity stress. Surprisingly, the Fv/Fm values seemed to not depend strictly on the cell densities of the samples, since the growth rate of samples grown at 36‰ (and the related total number of cells) was lower than samples grown at 20‰, which maintained a high efficiency until the end of the experiments, as did the 70‰ samples. Regarding molecular studies, various authors focused on specific genes, mainly coding antioxidants, such as catalase, superoxide dismutase, or glutathione peroxidase. Other works are on transcriptomic, proteomic, or metabolomic investigations [7]. 

Although *Chlamydomonas* spp. are known to live in multiple environments, including poles, and have often been considered good model species for adaptation studies, previous results showed strain-specific abilities to cope with stress [50]. The majority of the papers studying *Chlamydomonas* responses to salinity stress are focused on *C. reinhardtii* CC-503 (from Chlamydomonas Resource Center; http://chlamycollection.org/; accessed on 17 July 2024), *Chlamydomonas* W80 (isolated from the coastal area of Japan; [50]), *Chlamydomonas* ICE-L (isolated from Antarctic sea ice; [51]), and *C. nivalis* (especially for metabolomic and proteomic studies; [52,53,54]). Several differences have been observed among species and additional studies may be required to investigate the responses of *Chlamydomonas* strains inhabiting other environments. To our knowledge, this is the first study focusing on *Chlamydomonas* sp. CCMP225 (https://ncma.bigelow.org/CCMP225, accessed on 17 July 2024), isolated from Woods Hole, Massachusetts (USA), and exposed to salinity stress conditions. Regarding the experimental conditions, our work is the first to compare different growth phases, under two salinity conditions, in an estuarine *Chlamydomonas* species. Our experimental design allowed us to discriminate genes differentially expressed in the exponential or stationary phase at 20‰ and 70‰ salinity.

Considering previous studies on salinity stress exposure, we expected an increase in antioxidant enzymes (i.e., catalase, superoxide dismutase, ascorbate peroxidase, glutathione peroxidase, and glutathione reductase, as reviewed in Bazzani et al., 2021 [7]), the overexpression of the chaperone heat shock protein 70 (HSP70), and the calcium binding protein calmodulin. Surprisingly, we did not observe specific antioxidant enzymes’ activation. On the other hand, the high salinity stress response in our *Chlamydomonas* strain involved the down-regulation of calmodulin in the comparison S20_exp vs. S70_exp (with S70 being the control condition in this comparison), with a fold change of −3174, and the −3524 down-regulation of HSP70 in the same comparison. Hence, they were both more expressed at high salinity. HSPs are chaperones which have been previously reported to be essential for the correct protein folding and protein protection by stress induced by dehydration [55]. Overall, our results confirm that distinct species/strains may respond differently to stress conditions, and we expect that one may behave better than others to cope with adverse conditions.

From all comparisons of DEG analyses between the exponential and stationary phase, we could observe that there was the up-regulation of the following genes: Carbonic Anhydrase 2, 3-phosphoshikimate 1-carboxyvinyltransferase, chloroplastic, Chlorophyll a-b binding protein CP29.2, chloroplastic, UPF0225 protein VV1_2912, and Pyruvate dehydrogenase E1 component subunit beta-3, chloroplastic; and the down-regulation of the following genes: Serine/threonine-protein kinase fray1, Serine/threonine-protein kinase OSR1, Isoprenyl transferase, and Dehydrodolichyl diphosphate synthase 2. In particular, evolutionary conserved genes implicated in processes such as homeostasis [56], and metabolism were up-regulated. Among the down-regulated genes, there were the Serine/threonine protein kinases, which are involved in the phosphorylation of the OH group in serine and threonine, by regulating proliferation, apoptosis, and differentiation processes [57]. Additionally, enzymes involved in the correct folding of proteins such as isoprenyl transferase and dehydrodolichyl diphosphate synthase 2 [58] were also down-regulated. Other main differences were related to the expression of light-harvesting proteins, building the light-harvesting complex, and important for photoprotection, the expression of the carbonic anhydrase, known for maintaining acid–base homeostasis [59], and for catalyzing the reversible hydration of carbon dioxide [56]. Moreover, expression changes were detected for an intraflagellar transport protein known to play essential roles in the assembly and function of flagella (the intraflagellar transport protein 22, IFT22). Motility has a key role in survival [60]. Under stressful conditions, microalgae can undergo morphological changes like the loss of flagella. As reported in Silva et al., IFT22 regulates the amount of intraflagellar transport (IFT) particles distributed to the flagella, necessary for the transport from the cellular body of flagellar precursors in the flagellum [61]. Another aspect, highlighted by our results (as summarized in Figure 7), is an increase in lipid biosynthesis-related transcripts at high salinity. Similar effects have been also found for the plant *Catharanthus roseus* [62] and for *C. reinhardtii* [63]. In plants, it was also reported that salinity stress influences the transition metal homeostasis, with a reduction in transition metal uptake (e.g., Fe^2+^, Zn^2+^ and Cu^2+^) and an increase in their extrusion upon stress. However, the underlying mechanism is still unclear [64].

It has been reported that high salinity levels cause ionic, osmotic, and oxidative stress in both plants and microalgae, influencing the production of antioxidants and phenolic compounds [65]. Examples have been reported for the blue-green algae *Spirulina platensis* and the diatom *Phaeodactylum tricornutum* [65]. A recent study by Nezafatian et al. [66] showed the increased production of bioactive compounds from the marine microalgae *Tetraselmis tetrathele* under salinity and light stress exposure [66]. Recently, Saide et al. [67] showed that *Chlamydomonas* sp., the same alga used in the current study, showed anticancer properties after photoactivation, a process known as photodynamic therapy [67]. Here, we show that transcripts involved in the metabolism of anticancer molecules (i.e., 3-phosphoshikimate 1-carboxyvinyltransferase) were also differentially expressed under salinity stress. Additional studies on the bioactivity of this microalga in different salinity conditions will give new insights also on the possible discovery of marine natural products with applications for human pathologies. 

Overall, although additional studies are needed to better elucidate the molecular mechanisms behind the morphological and photosynthetic plasticity of this microalga, the data reported in this work will be of interest to both the ecological and biotechnological communities. This information will help predict and understand microalgal responses to potential climate change scenarios, and encourage biotechnological studies for human applications.

## 4. Materials and Methods

### 4.1. Microalgal Culturing

The microalga *Chlamydomonas* sp. (code CCMP225) was cultured in Guillard’s F/2 medium without silicic acid. The alga was cultured in control (normal medium with salinity at 36‰) and stressful condition (salinity at 20‰ and 70‰); 36‰ was selected as control because it is the total salinity range of the open ocean, and 20‰ and 70‰ were selected as extreme salinity stress conditions at which *Chlamydomonas* sp. have been previously found or tested, as reported by (Bazzani et al., 2021) [5,7]. The salinity was set by adding sea salts to filtered sea water or diluting filtered sea water. The experimental cultures were grown in 2-liter polycarbonate bottles, constantly mixed with soft air bubbling filtered through a 0.2 µm membrane, in a climate chamber at 18 °C on a 12:12 h light:dark cycle, and at 50 µE. Every experiment was performed in triplicate. Initial cell concentrations were approximately 5000 cells mL^−1^ for each replicate. Culture growth was monitored each day (for 19 days) by fixing 2 mL culture with lugol (final concentration of about 2%) and counted in a Bürker counting chamber under an Axioskop 2 microscope (Carl Zeiss GmbH). From the growth curves (cells/ml), we identified the exponential growth phase as the steepest portion of the curve, and derived the growth rate. To calculate the growth rate (K^10^), the cell concentration data are transformed into a natural logarithm (log_10_) and the linear regression factor is calculated on the exponential portion of the curve with Equation (1):

K^10^ = log(C_f_/C_i_). (t_f_−t_i_)^−1^ (1)

where K^10^ is the growth rate; C_f_ is the final concentration; C_i_ is the initial concentration; t_f_ is the upper limit of the interval time; t_i_ is the lower limit of the interval time; and C is the concentration of cells mL^−1^ in the stock culture.

To obtain the growth speed expressed as divisions day^−1^ (K_2_), we multiplied the K^10^ value by the constant 3.322, in order to obtain the number of divisions per day, calculated starting from data expressed in base 2 logarithm (log_2_) [68] with Equation (2):

K_2_ = K^10^. 3.322 (2)

where 3.322 is a conversion factor obtained from the ratio 1/log/ln 2 (2.3026/0.6931). 

During the cultivation, samples were collected for further analyses. To verify any morphological and morphometric differences of the cells under the different experimental conditions, the subsamples were collected and fixed, and analyzed with scanning electronic microscope (SEM). Fixed samples in lugol (about 2%) were stored in the fridge at 4 °C until further processing.

For RNA extraction and transcriptome sequencing (performed for the extreme salinity conditions 20‰ and 70‰ in the exponential Exp and stationary Stat phases), aliquots of 50 mL were sampled in the exponential phase (6 days) and in the stationary phase (12 days), for each salinity condition in triplicate, and centrifuged for 15 min at 4 °C at 1900× *g* (Eppendorf, 5810 R, Hamburg, Germany). For RNA preservation, 500 µL of TRIZOL© (Thermo Fisher Scientific, Waltham, MA, USA) were added to microalgal pellets, incubated for 2–3 min at 65 °C until completely dissolved, and frozen in liquid nitrogen. Then, samples were stored at −80 °C until the RNA extraction procedure [69]. 

The Fv/Fm values were determined using a Phyto_PAM (Walz, Effeltrich, Germany). All samples were acclimatized for 15 min in the dark before analysis to minimize the non-photochemical dissipation of excitation, and measurements were blank-corrected by filtering the sample through a 0.2 µm filter. For determining Fv/Fm, samples were illuminated with a saturating pulse following Maxwell and Johnson (2000). The Kruskal–Wallis test was used to compare the medians of the different treatments in order to define the significance of the differences observed between the samples [70]. 

### 4.2. RNA Extraction and Quality Check

RNA extraction was performed following the TRIZOL® (Thermo Fisher Scientific, Waltham, MA, USA) manufacturer’s instructions. RNA quantity and quality were evaluated as in Lauritano et al., 2015 [69] by using 2 µL per sample and measuring the absorbance at 260 nm and the 260/280 nm and 260/230 nm ratios by using Nano-Drop (ND-1000 UV–Vis spectrophotometer; NanoDrop Technologies, Wilmington, DE, USA). RNA samples were considered good when both ratios were approximately 2. RNA quality was also assessed on 1.5% agarose gel to check if the ribosomal bands were not degraded, as describe in Lauritano et al., 2015 [69].

### 4.3. RNA Sequencing, De Novo Assembly, and Differential Expression Analysis

Next-generation sequencing experiments were performed by Genomix4life S.R.L. (Baronissi, Salerno, Italy). RNA concentration in each sample was assayed with a Nanodrop One (Thermo Fisher Scientific, Waltham, MA, USA) and its quality assessed with the TapeStation 4200 (Agilent Technologies, Santa Clara, CA, USA). Indexed libraries were prepared from 700 µg/ea purified RNA with Illumina Stranded mRNA Prep according to the manufacturer’s instructions. Libraries were quantified using the TapeStation 4200 (Agilent Technologies, Santa Clara, CA, USA) and Qubit fluorometer (Thermo Fisher Scientific, Waltham, MA, USA), then pooled such that each index-tagged sample was present in equimolar amounts. The pooled samples were subject to cluster generation and sequencing using an Illumina NovaSeq6000 (Illumina, San Diego, CA, USA) in a 2x101 paired-end format.

The raw sequence files generated (.fastq files) underwent quality control analysis using FastQC [71] and the quality checked reads were trimmed with cutadapt. The high-quality reads from the sample were used as input to perform transcriptome assembly, with Trinity [71]. Trinity provides direct support for running the alignment-based quantification methods RSEM and eXpress, as well as the ultra-fast alignment-free method kallisto and wicked-fast salmon. We selected RSEM methods [23]. Trinity provides support for several differential expression analysis tools; we selected DeSeq2 package [25] with False Discovery Rate ≤0.05 for genes differentially expressed and isoforms differentially expressed. 

To perform the functional annotation and analysis of genome-scale sequence datasets, we used TransDecoder 5.5.0 to find coding regions within transcripts [72] and Trinotate 3.2.2 project for transcript functional annotation, which leverages both Trinity and TransDecoder as inputs [73]. TransDecoder identifies candidate coding regions within transcript sequences, such as those generated by de novo RNA-Seq transcript assembly using Trinity. TransDecoder identifies likely coding sequences based on the following criteria: (1) a minimum length open reading frame (ORF) is found in a transcript sequence; (2) a log-likelihood score similar to what is computed by the GeneID software is > 0; (3) the above coding score is greatest when the ORF is scored in the first reading frame as compared to scores in the other two forward reading frames; (4) if a candidate ORF is found fully encapsulated by the co-ordinates of another candidate ORF, the longer one is reported—however, a single transcript can report multiple ORFs (allowing for operons, chimeras, etc.); (5) a PSSM is built/trained/used to refine the start codon prediction; and (6) optionally, the putative peptide has a match to a Pfam domain above the noise cutoff score.

Trinotate is a comprehensive annotation suite designed for automatic functional annotation of transcriptomes, particularly de novo assembled transcriptomes, from model or non-model organisms. Trinotate makes use of a number of different well-referenced methods for functional annotation including homology search to known sequence data (BLAST+/SwissProt), protein domain identification (HMMER/PFAM), and leveraging various annotation databases (eggNOG/GO/Kegg databases). All functional annotation data derived from the analysis of transcripts are integrated into an SQLite database which allows fast efficient searching for terms with specific qualities related to a desired scientific hypothesis or a means to create a whole annotation report for a transcriptome. 

Gene ontology enrichment analysis was performed on genes differentially expressed that exceed the FDR and fold-change thresholds (FDR<=0.05, |FC|>=1.5). We used Trinotate to generate an annotation report, which includes top-matching blast matches from SwissProt and any corresponding gene ontology (GO) assignments from the TrEMBL/SwissProt databases. Then, the Bioconductor package GOseq was used to perform functional enrichment tests. The GOseq analysis was performed based on all up-/down-regulated genes separately. Venn diagram was carried out by using the Venn diagram web-tool (https://bioinformatics.psb.ugent.be/webtools/Venn/, accessed on 15 July 2024).

### 4.4. cDNA Synthesis and Reverse-Transcriptase–Quantitative PCR (RT-qPCR) Analysis

For RT-qPCR, five genes of interest between the most up- and down-regulated differentially expressed genes (DEGs) were selected: two transcripts for Adenylate cyclase, Chlorophyll a-b binding protein type member F3, chloroplastic, Probable inactive leucine-rich repeat receptor kinase XIAO, Carbonic anhydrase 2, and 3-phosphoshikimate 1-carboxyvinyltransferase, chloroplastic. Primers were designed using the software Primer3 version 4.1.0 (https://primer3.ut.ee/ accessed on 13 July 2024). The size of amplicon was kept in the range of 150–200 base pairs, the length of primers between 19–20 nucleotides, the melting temperature about 60 °C, and the CG content of about 50%. In Supplementary Table S7, primers sequences for all selected genes were reported. Actin, Ubiquitin, and Regulator of K+ conductance RCK1 (from Colina et al., 2019 [74]) were selected as possible reference genes. Then, 1 µg of RNA sample were retrotrascribed into complementary DNA (cDNA) using the iScriptTM cDNA Syntheis Kit (BIORAD, Hercules, CA, USA) following the manufacturer’s instructions. A dilution of 1:20 of cDNA was used as a template for RT-qPCR by using the ViiA7 real-time PCR system (Applied Biosystems, Waltham, MA, USA). PCR volume of each sample was 10 μL with 5 μL of PowerTrack^TM^ SYBR^TM^ Green Master Mix (Thermo Fisher Scientific, Waltham, MA, USA), 0.7 pmol/μL for each oligom and 1 μL of cDNA as template. To study the expression level for each gene of interest in the various conditions, we used the Relative Expression Software Tool (REST) [75]. The genes UBQ and RCK1 were used as reference for the analyses because they were assessed as most stable reference genes by the software Bestkeeper [76], NormFinder [77], and GeNorm [78] (by analyzing ACT, UBQ, and RCK1; [74]). Statistical analysis was performed by GraphPad Prism (version 8.1.2, GraphPad Software Inc., San Diego, CA, USA) by using a two-way analysis of variance (ANOVA) following by Tukey’s multiple-comparison test (*p*: 0.12 (not significant); 0.033 (*); 0.002 (**); and <0.001 (***)).

## Figures and Tables

**Table 1:** This table shows the total number of raw counts of genes and isoforms expressed in all samples. The abbreviation exp stands for exponential, stat for stationary, and rep for replicate. # stands for number.

Sample_Name	#Genes	#Isoforms
S20exp_rep1	47293	61173
S20exp_rep2	38171	49096
S20exp_rep3	42641	54014
S20stat_rep1	73803	97149
S20stat_rep2	76236	102698
S20stat_rep3	66162	91150
S70exp_rep1	74735	97753
S70exp_rep2	73391	96712
S70exp_rep3	71644	91918
S70stat_rep1	64904	85302
S70stat_rep2	68462	88701
S70stat_rep3	59995	79730

**Table 2:** This table reports the number of differentially expressed genes for each comparison and those identified with a false discovery rate (FDR) ≤0.05, in total, up-regulated (FC ≥ 1.5), or down-regulated (FC ≤ −1.5). The abbreviation “Exp” stands for exponential and “Stat” for stationary.

Treated_vs._CTRL	FDR ≤ 0.05	FC ≥ 1.5 & FDR ≤ 0.05	FC ≤ −1.5 & FDR ≤ 0.05
S20_Exp vs. S20_Stat	15094	6036	9040
S70_Exp vs. S70_Stat	2036	1060	903
S20_Exp vs. S70_Exp	17623	6809	10704
S20_Stat vs. S70_Stat	18926	10543	8322
Exp vs. Stat	2610	1627	977

**Table 3:** This table represents #GO (gene ontology) terms enriched, considering all up-regulated or down-regulated differentially expressed genes (DEGs), and considering, as comparison, S20_Exp vs. S20_Stat, S70_Exp vs. S70_Stat, S20_Exp vs. S70_Exp, S20_Stat vs. S70_Stat, and Exp vs. Stat, where the abbreviation “Exp” stands for exponential, “Stat” for stationary, and CTRL for control.

Treated_vs._CTRL	#GO Terms Enriched	
	Up DEGs	Down DEGs
S20_Exp vs. S20_Stat	33	1
S70_Exp vs. S70_Stat	0	0
S20_Exp vs. S70_Exp	13	0
S20_Stat vs. S70_Stat	13	17
Exp vs. Stat	35	0

**Table 4:** Five most up-regulated and down-regulated genes in *Chlamydomonas* sp. in differential expression analysis between S20_Exp vs. S20_Stat, where the abbreviation exp stands for exponential and stat for stationary.

Up-Regulated Genes	Fold Change	Down-Regulated Genes	Fold Changes
3-phosphoshikimate1carboxyvinyltransferase	+56.70	Adenylate cyclase	−6.54
Carbonic anhydrase 2	+51.19	Peptide-N(4)-(N-acetyl-beta-glucosaminyl)asparagine amidase	−6.22
UPF0225 protein VV1_2912	+48.34	Adenylate cyclase	−3.83
Pyruvate dehydrogenase E1 component subunit beta-3	+28.16	Ubiquitin carboxyl-terminal hydrolase 24	−2.83
Chlorophyll a-b binding protein CP29.2, chloroplastic	+16.72	Serine/threonine-protein phosphatase 6 regulatory ankyrin repeat subunit C	−2.78

**Table 5:** Five most up-regulated and down-regulated genes in *Chlamydomonas* sp. in differential expression analysis of S20_Exp vs. S70_Exp where the abbreviation exp stands for exponential and stat for stationary.

Up-Regulated Genes	Fold Change	Down-Regulated Genes	Fold Change
Chlorophyll a-b binding protein CP29.2, chloroplastic	+21.97	Serine/threonine-protein kinase fray1	−120.888
Chlorophyll a-b binding protein type member F3, chloroplastic	+15.02	Serine/threonine-protein kinase OSR1	−120.888
Carbonic anhydrase 2	+11.84	Isoprenyl transferase	−57.222
UPF0225 protein VV1_2912	+9.78	Dehydrodolichyl diphosphate synthase 2	−57.222
3-phosphoshikimate 1-carboxyvinyltransferase, chloroplastic	+8.05	Nitric oxide synthase	−31,74

**Table 6:** Five most up-regulated and down-regulated genes in *Chlamydomonas* sp. in differential expression analysis of S20_Stat vs. S70_Stat where the abbreviation exp stands for exponential and stat for stationary.

Up-Regulated	Fold Change	Down-Regulated	Fold Change
Probable inactive leucine-rich repeat receptor kinase XIAO	+217.457	Serine/threonine-protein kinase fray1	−34.91
Adenylate cyclase	+21.778	Serine/threonine-protein kinase OSR1	−34.91
Adenylate cyclase	+10.119	Isoprenyl transferase	−8.48
Cys-loop ligand-gated ion channel	+9.003	Dehydrodolichyl diphosphate synthase 2	−8.48
Intraflagellar transport protein 22	+6.971	Beta-carotene 3-hydroxylase, chloroplastic	−5.44

**Table 7:** Five most up-regulated genes in *Chlamydomonas* sp. in differential expression analysis of Exp vs. Stat where the abbreviation exp stands for exponential and stat for stationary.

Up-Regulated	Fold Change
Carbonic Anhydrase 2	+33.138
3-phosphoshikimate 1-carboxyvinyltransferase, chloroplastic	+11.175
Chlorophyll a-b binding protein CP29.2, chloroplastic	+9.657
UPF0225 protein VV1_2912	+9.569
Pyruvate dehydrogenase E1 component subunit beta-3, chloroplastic	+6.533

## Data Availability

Raw reads are available in the public database SRA (code PRJNA1096252).

## Supplementary Materials

The following supporting information can be downloaded at: https://www.mdpi.com/article/10.3390/md22080351/s1, Figure S1: Principal component analysis (PCA) of samples of exponential (Exp) and stationary (Stat) phases at 20‰ and 70‰ salinity; Table S1: Differential expressed genes in the comparison S20_Exp vs. S20_Stat; Table S2: Differential expressed genes in the comparison S70_Exp vs. S70_Stat; Table S3: Differential expressed genes in the comparison S20_Exp vs. S70_Exp; Table S4: Differential expressed genes in the comparison S20_Stat vs. S70_Stat; Table S5: Differential expressed genes in the comparison Exp vs. Stat; Table S6: Expression levels of selected genes of interest in *Chlamydomonas* sp. cultured in 20‰ and 70‰ salinity stress collected in exponential and stationary phases. Data are represented as log2 x-fold expression ratio ± SD (*n* = 3). The table also reports fold-change results of differential expression analyses of the RNA-seq for comparison; Table S7: The table shows the primers, forward and reverse, used for Real Time-PCR analyses, their abbreviation, and sequence code.
