# Supplementary material for: Salinity Stress Acclimation Strategies in Chlamydomonas sp. Revealed by Physiological, Morphological and Transcriptomic Approaches
Source: Mar Drugs. 2024 Jul 29;22(8):351. doi: 10.3390/md22080351 (PMC11355073; doi:10.3390/md22080351)

Figure S1: Principal component analysis (PCA) of samples of exponential (Exp) and stationary (Stat) phases at 20‰ and 70‰ salinity.

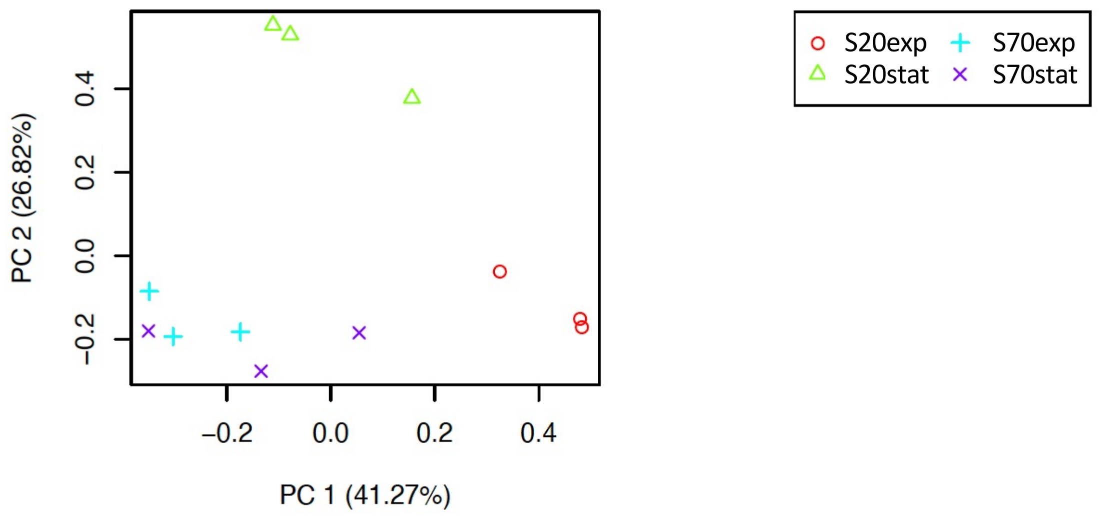

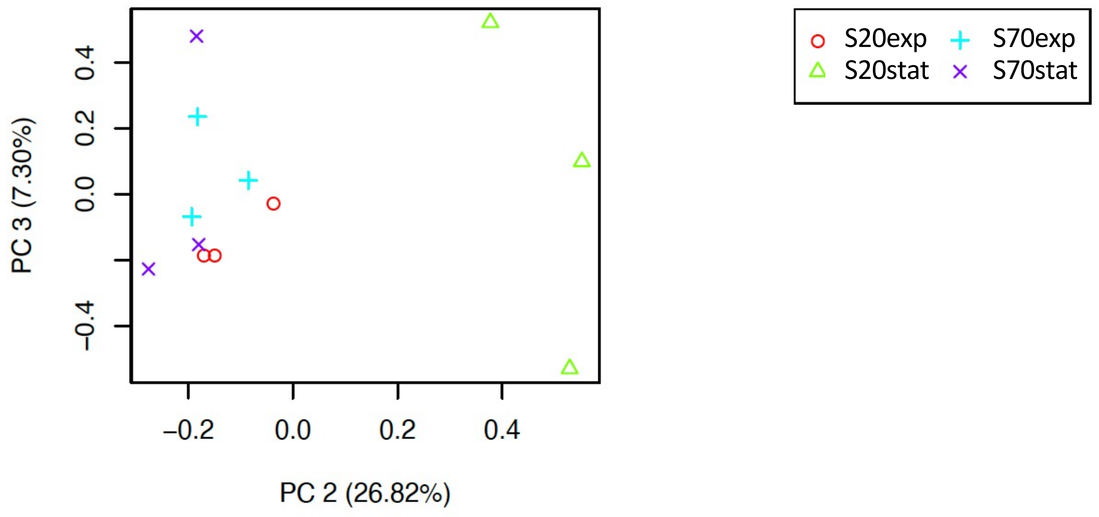

Supplement: Supplementary file 1 [file marinedrugs-22-00351-s001.zip › supplementary/Supplementary figure 1_PCA con didascalia.pdf]
