# Supplementary material for: Salinity Stress Acclimation Strategies in Chlamydomonas sp. Revealed by Physiological, Morphological and Transcriptomic Approaches
Source: Mar Drugs. 2024 Jul 29;22(8):351. doi: 10.3390/md22080351 (PMC11355073; doi:10.3390/md22080351)
Supplement: Supplementary file 1 [file marinedrugs-22-00351-s001.zip › supplementary/Supplementary table S6 rt.docx]

**Supplementary table 6:** Expression levels by reverse transcription quantitative PCR (RT-qPCR) of selected genes of interest in *Chlamydomonas* sp. cultured in 20 ‰ and 70 ‰ salinity stress collected in exponential (exp) and stationary (stat) phases. Data are represented as log_2_ x-fold expression ratio (*n* = 3). The table also reports fold change results of differential expression analyses of the RNA-sequencing (RNA-seq) for comparison.

| Gene | Fold change (RNA-seq) | DE comparison conditions (RNA-seq) | log_2_ x-fold ratio (RT-qPCR) | p value (RT-qPCR) |
| --- | --- | --- | --- | --- |
| AC | -6.54 | Down-regulated in S20_exp vs S20_stat | -2.781 | p=0.001 *** |
| CABF3 | +15.02 | Up-regulated in S20_exp vs S70_exp | +2.005 | p=0.001 *** |
| XIAO | +217.457 | Up-regulated in S20_stat vs S70_stat | +22.482 | p=0.003 ** |
| AC | +21.778 | Up-regulated in S20_stat vs S70_stat | +23.454 | p=0.001 *** |
| CA | +33.138 | Up-regulated in exp vs stat | +1.215 | p= 0.005 ** |

Abbreviations stand for: Adenylate cyclase (AC), Carbonic Anhydrase 2 (CA), Chlorophyll a-b binding protein type member F3, chloroplastic (CABF3); differential expression (DE), Probable inactive leucine-rich repeat receptor kinase XIAO (XIAO).
